# Supplementary material for: Immune responses in carp strains with different susceptibility to carp edema virus disease
Source: PeerJ. 2023 Jul 14;11:e15614. doi: 10.7717/peerj.15614 (PMC10351508; doi:10.7717/peerj.15614)
Supplement: Table S1 — Different letters indicate significant differences at p ≤ 0.05 between carp strains. [file peerj-11-15614-s001.docx]

|  | | | | | |
| --- | --- | --- | --- | --- | --- |
|  | **CEV Virus load, mean copies of viral DNA per 250 ng of (Gills)** | |  | **Replication CEV P4a mRNA normalized against 100000 copies of carp 40S ribosomal protein S11 (Gills)** | |
| **Fish** | **Day 6** | **Day 11** |  | **Day 6** | **Day 11** |
| Koi  Mean  Median  SD | 6.41E+04**^a^**  4.44E+04  6.90E+04 | 5.65E+04^a^  6.45E+03  1.04E+05 |  | 4.28E+03 ^a^  2.23E+03 | 1.36E+03 ^ab^  1.43E+02 |
| PS  Mean  Median  SD | 1.37E+04^ab^  8.94E+03  1.64E+04 | 3.17E+05^a^  1.61E+04  6.09E+05 |  | 1.49E+03 ^a^  1.49E+03 | 4.95E+03 ^a^  1.08E+03 |
| Rop  Mean  Median  SD | 8.66E+02 ^ab^  2.50E+01  1.70E+03 | 1.75E+03 ^a^  4.92E+02  2.84E+03 |  | 2.18E+02 ^b^  - | 1.37E+02 ^ab^  6.00E+00 |
| AS  Mean  Median  SD | 1.50E+02 ^b^  1.14E+02  1.33E+02 | 4.08E+02 ^a^  3.61E+02  3.76E+02 |  | 1.00E+01 ^b^  - | 5.00E+00 ^b^  5.00E+00 |
|  | | | | | |
