# Supplementary material for: Immune responses in carp strains with different susceptibility to carp edema virus disease
Source: PeerJ. 2023 Jul 14;11:e15614. doi: 10.7717/peerj.15614 (PMC10351508; doi:10.7717/peerj.15614)
Supplement: Table S2 — Different letters indicate significant differences at p ≤ 0.05 between carp strains. [file peerj-11-15614-s002.docx]

|  | | | | | |
| --- | --- | --- | --- | --- | --- |
|  | **CEV Virus load, mean copies of viral DNA per 250 ng of (Gills)** | |  | **Replication CEV P4a mRNA normalized against 100000 copies of carp 40S ribosomal protein S11 (Gills)** | |
| **Fish** | **Day 6** | **Day 11** |  | **Day 6** | **Day 11** |
| Koi  Mean  Median  SD | 1.52E+05 ^a^  1.13E+05  1.29E+05 | 1.25E+06^a^  1.03E+06  8.11E+05 |  | 5.14E+02 ^a^  4.77E+02 | 3.08E+03 ^a^  3.06E+03 |
| PS  Mean  Median  SD | 2.58E+03 ^bc^  2.83E+03  1.78E+03 | 5.62E+02 ^b^  4.61E+02  2.85E+02 |  | -  - | -  - |
| Rop  Mean  Median  SD | 4.73E+03 ^b^  4.78E+03  2.56E+03 | 1.69E+03 ^b^  4.65E+02  2.54E+03 |  | -  - | -  - |
| AS  Mean  Median  SD | 6.04E+02 ^c^  5.55E+02  5.20E+02 | 4.36E+02 ^b^  3.94E+02  4.26E+02 |  | <1  - | <1  - |
|  | | | | | |
